# Frequency evaluation of genes encoding siderophores and the effects of different concentrations of Fe ions on growth rate of uropathogenic *Escherichia coli*

**Published:** 2016-12

**Authors:** Behnoush Khasheii, Shaghayegh Anvari, Ailar Jamalli

**Affiliations:** 1 Faculty of Medicine, Golestan University of Medical Sciences, Gorgan, Iran; 2 Infectious Diseases Research Center, Golestan University of Medical Sciences, Gorgan, Iran

**Keywords:** *Escherichia coli*, Siderophore, Aerobactin, Salmochelin, Yersiniabactin, Fe (II) supplements

## Abstract

**Background and Objectives:**

Bacteria need iron for growth and most of them can actively acquire Fe ions using especial iron-chelating proteins which named siderophores. We aimed to determine the frequencies of *iucA, iroN* and *irp2* genes in the uropathogenic *Escherichia coli* (UPEC) isolates. We also analyzed the effects of siderophore genes beside iron supplements on growth rate of the isolates.

**Materials and Methods:**

Totally, 170 *E. coli* strains were isolated from urinary tract infections and the presence of 3 siderophore genes were analyzed using PCR among them. Three final concentrations of 0.1, 0.5 and 1 mMFe(II) and Fe(III) ions were made in M9 broth medium. Inoculated cultures were incubated at 37°C for 33 hours and bacterial density in the suspension was measured with 1 hour intervals using spectrophotometer.

**Results:**

The frequency of *iucA, iroN* and *irp2* genes among 170 UPEC isolates were 29 (17.1%), 52 (30.6%) and 116 (68.2%), respectively. In addition, Our findings showed that Fe(II) supplements had significantly higher promoting effects on UPEC growth rate almost in all of the three applied concentrations (0.1, 0.5 and 1 mM) compared to the control group (*P*<0.0001). Differences between Fe(III) supplemented groups and the controls were statistically significant when 1 mM concentration was added into the medium (p<0.05).

**Conclusion:**

*irp2* gene probably plays a major role in the pathogenesis of UPEC strains. Promoting or inhibitory effects of iron on bacterial growth mainly depend on the iron concentration in the culture medium however different siderophores have different potentials for capturing and assimilation of Fe ions by the bacteria, especially inside the host cell.

## INTRODUCTION

Iron is an essential element for supporting growth and development of any living microorganism including *E. coli*. Iron, as a unique catalyzer, contributes to the initiation and progression of many biological pathways including tricarboxylic acid (TCA) cycle, electron transport chain (ETC), oxidative phosphorylation, nitrogen fixation and biosynthesis of many aromatic compounds (1). Bacterial growth and development need at least 10^−6^ M iron (2). On the other hand, the higher iron concentrations may be cytotoxic and show bactericidal or antimicrobial effects on the microorganisms. Association between iron concentration and microbial infection has been demonstrated by many scientists through empirical studies in humans or laboratory animals (3). Mammals usually employ an efficient defense strategy against bacterial pathogens such as high affinity iron-chelating molecules (like transferrin and lactoferrin). To scavenge free iron ions, these molecules will be released which lead to deprivation of invader microorganism from accessing the ions (4). In contrast, during urinary tract infections, uropathogenic *E. coli* (UPEC) strains employ different strategies including synthesis of several high affinity and low molecular weight proteins which are named siderophores (1). Siderophores belong to a chemically diverse family of molecules produced by many pathogenic and non-pathogenic bacteria to chelate iron ions (5). Siderophores are produced and secreted into the surrounding environment, have higher affinity to ferric than ferrous ions. Ferric-bound siderophore is captured and internalized by the bacteria using specific outer membrane receptors (1). *E. coli* strains may synthesize 4 types of siderophores named enterobactin, salmochelin, yersiniabactin and aerobactin. Many non-pathogenic *E. coli* strains were only capable for synthesizing enterobactin siderophore which is encoded by *ent-fes-fep* gene cluster (6). However, UPEC strains are also able to synthesize siderophores other than enterobactin which help bacteria for better survival in the iron-limited conditions like urinary tract. Bacterial aerobactin is encoded by *iuc* DBAC gene cluster whereas the salmochelin is encoded by *iroA* gene cluster and synthesis of yersiniabactin is encoded by high pathogenicity island (HPI) and *irp2, irp1,* ybtSETUXPQA gene clusters (7–9). Several studies have demonstrated the significant associations between aerobactin synthesis and certain infectious conditions like pyelonephritis and cystitis (10). Further surveys of female patients with recurrent urinary tract infections have revealed significant higher frequency of salmochelin and aerobactinsiderophore expression in isolated bacterial strains and these siderophores were considered as the main factors highly associated with recurrent infections and drug resistance (11). Current study was designed to survey the effect of two or three valent iron ions concentrations on the growth rate of UPEC strains isolated from urinary tract infections. We also determined the frequency of genes encoding aerobactin, salmochelin and yersiniabactin siderophores among isolated strains.

## MATERIALS AND METHODS

### 

#### Sample collection and bacterial identification

A cross-sectional descriptive analytic study has been done. During the study period, mid-stream urine samples were collected from suspected urinary tract infections from patients who admitted to university dependent healthcare centers in Gorgan, center of Gholestan province (North of Iran) or samples from outpatients with the same condition referred to the private sector medical laboratories. Standard conventional procedures were applied for isolation and identification of enterobacteriaceae including IMViC biochemical tests and finally a total of 170 *E. coli* strains were isolated in M9 broth media after 24 h incubation at 37°C.

#### Genomic DNA extraction and PCR amplification

Boiling method was used genomic DNA extraction according to a previously published study (12). To examine the presence of *IucA, IroN* and *Irp2* genes among *E. coli* isolates, we used 3 specific pairs of primers (Table 1). PCR conditions were set according to the study conducted by Karimian et al (13) and the products were visualized on 1.5% agarose gel.

**Table 1.** Primer pairs for PCR amplification of *IucA, IroN* and *Irp2* genes in uropathogenic *E. coli* isolates

**Genes**	**Primer sequence (5’-3’)**	**PCR product (bp)**	**Ref**
*iucA*	F: 5’-atgagaatcattattgacataattg-3’ R: 5’-ctcacgggtgaaaatatttt-3’	1482	(14)
*iroN*	F: 5’-aagtcaaagcaggggttgcccg-3’ R: 5’-gacgccgacattaagacgcag-3’	665	(15)
*irp2*	F: 5’-aaggattcgctgttaccggac-3’ R: 5’-Aactcctgatacaggtggc-3’	413	(16)

### Culture of bacteria in media supplemented with Fe(II) or Fe(III) ions:

#### Selection of representative strains

Based on PCR results, e.g., presence or absence of *irp2, iroN,* and *iucA* genes in the bacteria, 170 UPEC isolates were divided into 8 groups and one representative strain from each group were selected for evaluating iron element effects on bacterial growth rate and correlating the rate with the types of siderophores produced by the bacteria. Eight representative strains and details of their genotypes were presented in Table 2.

**Table 2.** Based on PCR results, eight representative strains were selected to study iron effect on microbial growth rate.

**Strain name**	***iucA***	***iroN***	***irp2***
E-132	+	−	−
E-331	−	−	+
E-131	+	+	−
E-462	−	+	−
E-335	−	+	+
E-477	+	−	+
E-66	+	+	+
E-501	−	−	−

#### Preparation of Fe^2+^ and Fe^3+^ stock solutions

For preparation of 20 mM Fe^2+^ stock solution, 556 mg ferrous sulfate (FeSO_4_, 7H_2_O, Merk, Germany) was weighed and dissolved in distilled water (final volume: 100ml). For preparation of 20 mM Fe^3+^ stock solution, 541 mg ferric chloride (FeCl_3_, 6H_2_O, Merk) was weighed and dissolved in DW (final volume 100 ml). Both stock solutions were filter sterilized (using a 0.4 micron pore size filter).

#### Culture media

M9 broth medium (45-63011-500G-F, Fluka, Germany) supplemented with 0.5% glucose was used for supporting bacterial growth. The medium was prepared according to the manufacturer recommendations and autoclaved at 121°C for 15 min for sterilization.

#### Plotting growth curve in the absence and presence of iron supplements

Representative strains were cultured in M9 broth medium and incubated at 37°C for 24 hours. One ml of the suspension was inoculated into 4 ml freshly prepared M9 broth media and incubated again at 37°C until the turbidity of the suspension was reached to 0.5 McFarland standard turbidity (1.5 × 10^8^ cells/ml). A 2.5 ml sample from each standardized bacterial suspension was added to 50 ml of M9 broth media and serial dilution were prepared. Finally a working suspension containing 10^6^ bacterial cells per ml was resulted. Then 250, 1250 and 2500 μl from the two stock iron solutions were added to the bacterial suspensions (final iron concentrations equal to 0.1, 0.5 and 1 mM). Turbidity of these tubes was measured and considered as the zero point for bacterial growth curve prediction. A series of control tubes containing media plus bacterial suspension, but omitting iron addition step was included. Blank tube series were also included in the experiment. The blanks had M9 broth media and iron but not bacterial strains. All tests, control, and blank tubes were incubated at 37°C with shaking. Optical density (OD) of tubes was read at 600 nm with one hour intervals using a spectrophotometer and the resulting data was used for plotting growth curve of the each bacterial isolate (17–19). To achieve the highest accuracy, all experiments were repeated two or more times. For plotting growth curves log10 values of bacterial densities were presented on Y axis and the time points, as hours, went on X.

#### Statistical analysis

SPSS software (v. 16.0) was used for statistical analysis. ANOVA was used for comparing the mean values between all studied groups. Tukey test was used for testing equality of variances. Two independent-samples t-test was used for binary comparisons. P value less than 0.05 was considered as statistically significant.

## RESULTS

### 

#### Distribution of siderophore genes

**Fig. 1.**
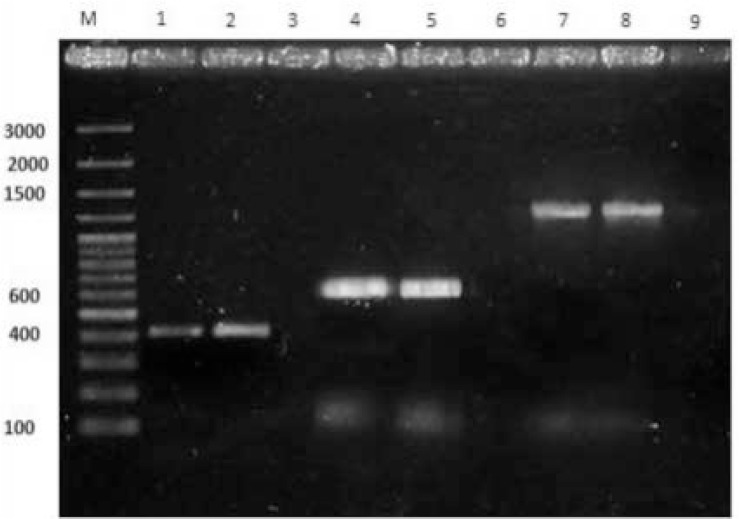
PCR amplification of *irp2, iroN* and *iucA*. M: DNA molecular weight marker, lanes 1, 2(*Irp2*), 4, 5(*IroN*), 7, 8(*IucA*): PCR aproducts from positive strains relating to the three siderophore genes. lanes of 3,6,9: Negative strains lacking any of the three detected siderophore genes.

PCR amplification of three siderophore genes among 170 UPEC isolates revealed that *iucA, iroN* and *irp2* genes were present among 29 (17.1), 52 (30.8%) and 116 (68.2%) strains, respectively (Fig. 1). In addition, 38 isolates (22.4%) did not possess any of the studied genes and 7 isolates (4.1%) had all of the 3 genes. Four isolates (2.4%) had only aerobactin gene (*iucA*), 60 isolates (35.3%) were only positive for yersiniabactin gene (*irp2*) and 10 isolates (5.9%) only represented salmochelin gene (*iroN*). Two isolates (1.2%) carried both aerobactin and salmochelin genes (*iucA/iroN*). Sixteen isolates (9.4%) showed positivity for both aerobactin and yersiniabactin genes (*iucA/irp2*). Finally, 33 isolates (19.4%) had both of salmochelin and yersiniabactin genes (*iroN/irp2*).

#### Effects of supplemental Fe^2+^/Fe^3+^ ions on bacterial growth

**Fig. 2.**
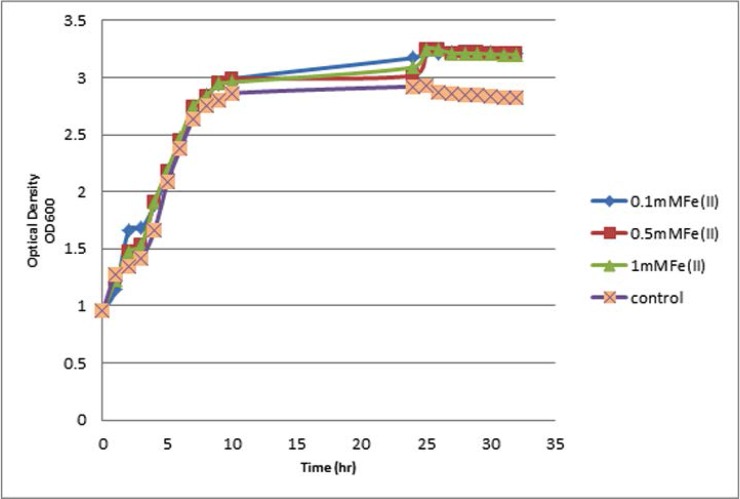
Growth curve of *E. coli* E-331 strain harboring yersiniabactinsiderophore in the presence or absence of Fe(II) ions

Incubation of selected strains in broth media supplemented with 0.5% glucose and three distinct concentrations of Fe^2+^ or Fe^3+^ ions (0.1, 0.5 and 1 mM) and continuous monitoring of cellular density in both test and control groups revealed that strains harboring siderophores were better survived and regenerated in all three concentrations of iron ions. In addition comparing the Fe^2+^ ion supplementation effects on the bacterial growth rate using ANOVA statistical test clearly showed that there were obvious differences between test and control cultures regarding cell growth rate. Supplementing the media with Fe^2+^ represented growth promoting effects on tested bacteria nearly in all three concentrations which represented statistically significant differences (*P*<0.0001) (Fig. 2).

**Fig. 3.**
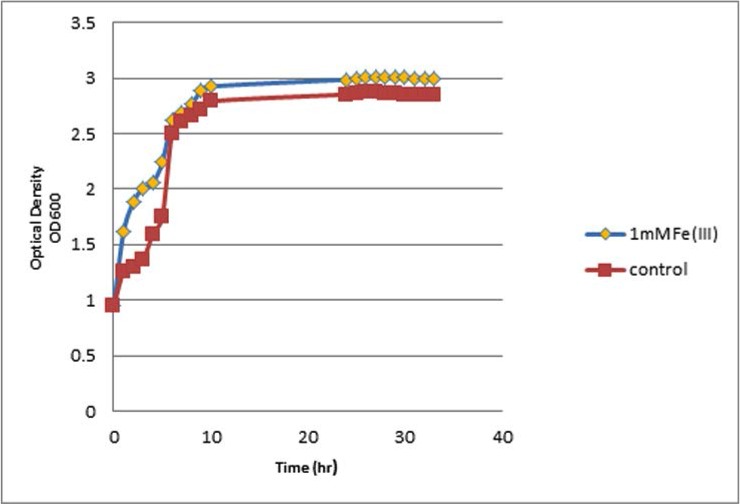
Growth curve of *E. coli* E-131 strain harboring both aerobactin and salmochelinsiderophores in the presence or absence of Fe(III) ions

The effects of different Fe^3+^ ion concentrations revealed that Fe^3+^ ions at concentrations of 0.1, 0.5, and 1 mM have increased growth effects on the bacterial strains; however, bacteria cultured in media supplemented with 1 mM Fe^3+^ ions exhibited a statistically significant increase in growth rates, when compared with the control cultured with no iron supplements (Tukey test, *P*<0.05) (Fig. 3). More importantly, our findings showed that bacteria cultured with Fe^2+^ supplementation had higher growth rates as compared with those cultured with Fe^3+^ supplementation (*P*<0.0001, Table 3).

**Table 3.** Compare statistical effect Fe^2+^/Fe^3+^ ions on productivity growth of strains according to the type of siderophore

**Strian**	**Fe^3+^**			**Fe^2+^**		

	**1mM**	**0.5mM**	**0.1mM**	**1mM**	**0.5mM**	**0.1mM**
E-132 (*IucA*)						
E-131 (*IroN-IucA*)						
E-331 (*Irp2*)						
E-462 (*IroN*)						
E-335 (*Irp2-IroN*)		*P* value=0.0001			*P* value=0.05	
E-477 (*IucA-Irp2*)						
E-66 (*IroN-IucA-Irp2*)						
E-501 (Non siderophor)						

#### The effect of siderophore types on bacterial growth in the presence of Fe^2+^/Fe^3+^ ions

Among selected strains cultured in the presence of Fe^2+^ ion, E-331 strain containing *irp2* gene had the highest growth rate in the presence of 0.1, 0.5 and 1 mM Fe^2+^ followed by E-66 strain containing all three identified siderophore genes (*iucA, iroN* and *irp2*). E-131 strain containing salmochelin and aerobactin genes also showed higher growth rate in the presence of 1 mM concentration of Fe^2+^.

Among selected strains cultured and supplemented with Fe^3+^ ions, E-335 strain harboring yersiniabactin and salmochelin genes had the highest growth rate in the presence of 0.1, 0.5 and 1 mM Fe^3+^. In the presence of 1 mM Fe^3+^, the highest bacterial density belonged to E-132 strain represented aerobactin gene (*iucA*) followed by E-131 strain containing aerobactin and salmochelin genes (*iucA/iroN*). In general, all bacterial control samples lacking iron supplementation showed obvious delay in growth compared to the supplemented media.

## DISCUSSION

Access to iron is of crucial importance for bacterial infections. The invading microbes often have to overcome the iron-limiting conditions imposed by the presence of high affinity iron-binding proteins in the body fluids of vertebrate hosts. UPEC strains, especially during urinary tract infections, implement different strategies for efficient iron acquisition from the iron-limited surrounding environment. One of the important strategies for survival and growth of bacteria in the environment is the synthesis of low molecular weight, high affinity iron-chelating agents named siderophores. Hitherto et al. has shown that UPEC strains may express aerobactin, salmochelin or yersiniabactin siderophores during the infection. Siderophores are usually bound to free Fe(III) ions and are also capable to dissociate ions from host-related iron-chelating molecules like transferrin, lactoferrin or haptoglobin. Finally, Fe(III)-siderophore molecules are recaptured from the environment and internalized by the bacteria using receptor-mediated mechanisms (1–4). Our findings showed that among *E. coli* strains isolated from urinary tract infections, the most frequently detected siderophore gene is *irp2* (68.2%) which encodes for yersiniabactin. This finding is in agreement with the results published by Abdi et al. in which 89% of isolated UPEC strains originated from urinary tract infections contained *irp2* gene positive (20). In the study conducted by Karimian et al. different frequencies were observed for these three siderophore genes among UPEC strains isolated from patients admitted to the emergency section of a university hospital in Tehran. The reported frequencies for *iucA, iroN* and *irp2* genes in this study were 10.6, 42.3 and 11.4%, respectively (13). Which are clearly different from our findings. The differences may be attributable to the different geographical locations. Momtaz et al. investigated frequency of *irp2* gene among 123 UPEC strains isolated from urinary tract infections of Iranian patients and showed that 14 out of 123 strains (11.4%) had the gene sequence in their genomic DNA (21). Nateghi et al. also analyzed the frequency of *irp2* and *iucD* genes among 100 UPEC isolates from Iranian patients and the frequencies were 33 and 0%, respectively (22). In 2014, Ali-Abdi et al. found that 40 and 83.6% of UPEC strains isolated from urinary tract infections of Iranian patients had *iroN* and *iucA* genes, respectively (23), which is clearly higher compared with our results. In 2011, Usein et al. studied the frequency of *irp2* and *iucC* genes among 93 *E. coli* strains isolated from vaginal fluid samples of Romanian women and found that the frequency of these genes was 85 and 51%, respectively (24). Inconsistent with our results, frequency of *irp2* gene was high (85 vs. 68.2% in our study). In a comparative study, Bauer et al. analyzed the frequency of *iroN* gene among *E. coli* strains isolated from urinary tract infections as well as feces of American patients and the results showed that 28 out of 74 UTI isolates (38%) and 34 out of 184 feces isolates (18.5%) had *iroN* gene (25). *iroN* gene frequency in this study was in close consistency with our results (38 vs. 30.8% in our study). In another study from Brazil, the frequency of *iucD* gene among 162 UPEC strains isolated from cystitis patients was 27.8percnt; (26) which is very close to our finding.

Several studies have been conducted to explore the effect of iron supplementation on bacterial growth. Kalantari et al. evaluated the influence of iron on growth rate of *E. coli* in nutrient broth as the culture medium (17). Obvious reduction in bacterial growth rate was reported in the presence of 1 mM Fe^+3^ or 0.5 mM Fe^+2^ compared with the control cultures without any supplemented iron. In addition, bacterial growth was totally inhibited in the presence of 1 mM Fe^+2^ (17). In the present study which was carried out using M9 broth medium, 8 UPEC strains containing different combinations of siderophore genes, iron addition not only had no toxic (inhibitory) effect but also showed promoting effect on bacterial growth even in the presence of up to 1 mM Fe^+2^ or Fe^+3^ ions. Although, both Fe^+2^ or Fe^+3^ ions had positive effects on the bacterial growth, however, the effect is further prominent in cultures supplemented with Fe^+2^ ions in comparison with matched cultures supplemented with Fe^+3^ and the differences were statistically significant. A study conducted by Chatterjee et al. the effect of iron oxide nano particles on the growth kinetics of *E. coli* was investigated in India. LB broth media supplemented with 50, 100, 150 and 200 μg/ml of the nano particles (Fe_3_O_4_) were used for supporting bacterial growth. In this experiment, iron nano-particles showed notable inhibitory effects on the growth of the exposed strains and the time needed for reaching to logarithmic growth phase of the bacteria was gradually decreased when the particle concentration in the medium was harmonically increased. In addition, bacterial growth was totally inhibited in higher concentrations of the nano particles (27). Another experiment Kalantari et al. compared the effect of ferric and ferrous ions on the growth of two bacterial species, *Bacillus cereous* and *E. coli.* The study showed that 11.2 mg/l concentration of ferrous or ferric ions in the culture medium could completely cease the growth of *E. coli* and *B. cereous*. In addition, 5.6 mg/l concentration of these two ions led to 48.4% reduction in the growth of *B. cereous* after 3.5 hours of incubation but did not show any inhibitory effect on the growth of *E. coli* strains. This study emphasized on the toxic effect of high concentrations of iron ions for these two bacteria (18). During the last decade, studies have demonstrated that pathogenic *E. coli* strains had higher iron requirements than environmental isolates. It has also been demonstrated that pathogenic strains isolated from human infections are able to express higher levels of siderophores in both iron-limited and iron-enriched conditions (28, 29).

Our study showed all the eight isolated UPEC can grow at higher rate in Fe^2+^ and Fe^3+^ concentrations including 0. 1, 0.5 and 1mM, regardless of siderophore genes. Nonetheless isolated UPEC bearing yersiniabactin and aerobactin siderophore genes has the highest growth in compare with other siderophore. This finding suggests that the higher growth rate of this to UPEC may be result in higher affinity of this siderophore to iron in compare with other siderophore. a hypothesis that should be checked in the future experiments.

## CONCLUSION

As for the highest frequency of *irp2* gene, encoding for yersiniabactin siderophore, observed among 170 UPEC isolates in the current study, it seems that *irp2* gene plays a major role in the pathogenesis of the bacteria and as a potential virulence factor which may assure the bacterial survival in the iron-limited conditions. Iron is an important element in the culture media which can promote or inhibit bacterial growth based on its concentrations. We also found that different siderophores have different potentials in capturing and assimilation of Fe ions by the bacteria.
